# Thermal, Spectroscopy and Luminescent Characterization of Hybrid PMMA/Lanthanide Complex Materials

**DOI:** 10.3390/ma14123156

**Published:** 2021-06-08

**Authors:** Małgorzata Gil-Kowalczyk, Renata Łyszczek, Anna Jusza, Ryszard Piramidowicz

**Affiliations:** 1 Laboratory of Optical Fibers Technology, Institute of Chemical Sciences, Faculty of Chemistry, Maria Curie-Skłodowska University, M. Curie-Sklodowska Sq. 5, 20-031 Lublin, Poland; 2 Department of General and Coordination Chemistry and Crystallography, Institute of Chemical Sciences, Faculty of Chemistry, Maria Curie-Skłodowska University, M. Curie-Sklodowska Sq. 5, 20-031 Lublin, Poland; renata.lyszczek@poczta.umcs.lublin.pl; 3 Institute of Microelectronics and Optoelectronics, Warsaw University of Technology, Institute of Microelectronics and Optoelectronics, Koszykowa 75, 00-662 Warsaw, Poland; anna.jusza@pw.edu.pl (A.J.); ryszard.piramidowicz@pw.edu.pl (R.P.)

**Keywords:** lanthanide complexes, spectroscopy, optical fiber

## Abstract

Novel hybrid materials based on the poly(methyl methacrylate) (PMMA) matrix and lanthanide(III) carboxylates Eu:2,6-DClB and Tb:2,6-DClB were synthesized and carefully analyzed in the context of their potential application in optically active polymer-based optical fibers. To determine the usefulness of the obtained materials, a careful thermal, mass spectroscopy, and optical characterization was performed, focusing on the features critical for the technology of optical fiber processing. In addition, the luminescent features of both lanthanide complexes and the resulting hybrid composites were carefully investigated to identify the processes responsible for light emission and to analyze the influence of the PMMA host on light emission intensity and spectral characteristics. The obtained results showed that both lanthanide carboxylate complexes exhibited intense luminescence in the red and green spectral range, typical of europium and terbium dopants, and that those features were well preserved after introducing them into the PMMA polymer. Thermal analysis also proved that introducing the luminescent additives did not significantly affect the thermal properties of both hybrid materials, thus enabling further processing into the form of optical fibers.

## 1. Introduction

The design and synthesis of new luminescent materials continuously attract tremendous attention due to the wide spectrum of their applications in modern photonic technologies, medicine, sensing, fiber-optic and free-space communication, solar energy conversion, etc. [1,2,3,4,5,6]. The doping of lanthanide ions into inorganic matrices, such as metallic oxides and oxysalts, is traditionally an efficient method to obtain luminescent materials of excellent performance [1,2,3,4,5,6,7,8].

Although various crystals and glasses doped with lanthanide ions are commonly deployed in many fields of modern photonics, there is still plenty of room for introducing new solutions, offering features extending their application range and/or lowering the manufacturing and exploitation cost. It seems that, at least, one of the major constraints of purely inorganic hosts is the challenge of processing such rigid compounds [5], which might be easily solved by introducing optically active dopants into more “flexible” matrices such as polymers. Apart from excellent flexibility, they also offer attractive features such as mechanical strength, ease of processing, and low manufacturing cost, outperforming their inorganic competitors. The combination of structurally diverse components (i.e., polymeric matrix and luminescent additive) may lead to the formation of hybrid materials of unique performance parameters benefiting from features inherent to both elements. The main advantage of such an approach is the possibility of precisely tailoring the desired functionality of the resulting material via the suitable choice of constituents that carry specific properties, thus paving the way to an almost unlimited range of applications [9,10,11,12,13].

For the manufacturing of hybrid materials based on the polymeric matrices compounds, such as poly(vinylpyrrolidone) (PVP), poly(methyl methacrylate) (PMMA) or epoxy resin based on poly(ethylene oxide) (ER-PEO) are commonly used. Poly(methyl methacrylate) belongs to the most explored polymeric matrices due to the fact of its excellent mechanical and optical properties as well as low cost. These features make this polymer the material of choice for the fabrication of optical displays and devices, solid-state lighting materials, and optical fibers [8,14,15,16,17,18,19]. Moreover, additionally important are carbonyl groups present in the structure of PMMA that may help form covalent bonds with other components of hybrid materials such as optically active lanthanide complexes.

Lanthanide complexes have recently been widely explored not only due to the fact of their fascinating crystalline structures but also due to the fact of their specific luminescent properties; they are characterized by sharp emission lines characteristic for intra-configurational optical transitions (except for La(III) and Lu(III)) in the broad spectral range extending from ultraviolet (UV) to infrared (IR), long fluorescence lifetimes, and large Stokes shift. Especially attractive are lanthanide complexes in which organic ligands play the role of sensitizers of lanthanide luminescence. The organic ligands coordinating the lanthanides efficiently absorb UV radiation and transfer absorbed energy to the lanthanide ions acting as a sort of antennas. This process significantly enhances emissions from lanthanide ions that typically suffer from weak light absorption and emission properties, as the transitions between 4f^n^ configurations of Ln(III) are forbidden by Laporte’s rule and their direct excitation is inefficient [18,20,21].

The unique luminescent properties of organic lanthanide complexes make them ideal dopants for different types of polymeric matrices. Lanthanide complexes incorporated in the matrix may be physically or chemically bonded with the polymeric framework. It is commonly known that the high coordination number (8 or 9) of lanthanide ions in complexes is saturated not only by donor atoms from organic ligands but also solvent molecules. During embedding of a lanthanide complex into a polymeric matrix, the substitution of solvent molecules by donor atoms from functional groups of the polymeric framework can take place [22,23]. Such a process leads to the formation of coordination bonds between the polymeric host and lanthanide centers. Components of hybrid materials are very often bonded via weaker non-covalent bonds such as Van der Waals interactions, π-stackings, or hydrogen bonds [24].

Among many investigated materials, lanthanide complexes based on the carboxylate ligands have attracted great attention as luminescent dopants into hybrid materials. One of the main advantages of such complexes is their high thermal stability, enabling thermal processing at temperatures up to 400 °C. When introduced into the polymer matrices, their thermal and mechanical strengths do not dramatically change, and this may result in the enhancement of the luminescent properties of the metal complex [25,26].

This work deals with novel hybrid materials based on the PMMA matrix and lanthanide(III) carboxylates. The dimeric europium(III) and terbium(III) complexes with the 2,6-dichlorobenzoate ligand were used as luminescent dopants introduced into the PMMA matrix to develop and investigate the properties of such a hybrid material. Particular emphasis was given to the investigation of the thermal behavior of the free components and obtained hybrid materials to determine the effect of the addition of lanthanide complexes on the thermal stability and pathways of thermal decomposition of the received materials. The research also focused on the identification of volatile products of heating in the inert atmosphere of output components and synthesized materials.

The main such careful thermal and spectroscopic analysis was the assessment of the usefulness of investigated materials in optical fiber technology. Typically, before the material is used to make a preform from which the optical fibers are drawn, it is subjected to such thermal analysis. These analyses provide a lot of information, among which one of the most important is amount of mass loss. Its value defines the temperature limit, which cannot be exceeded during the polymer optical fiber drawing procedure without the risk of a change in the polymer structure, which in turn would lead to a large scattering of light in the final product. In the following examples, PMMA was used as the polymer matrix. Based on previous data [27], we can conclude that poly(methyl methacrylate) obtained in the Laboratory of Optical Fibers Technology is stable up to a temperature of approximately 270 °C, with only 2 wt.% of mass loss. This information is important, as the fiber drawing process for materials based on PMMA is conducted in the temperature range of approximately 200–220 °C. On this basis, we concluded that it is necessary to use materials that, up to this temperature, will not show any mass loss or where the loss will be negligible. Apart from thermal analysis, the luminescence properties of the lanthanide complexes and synthesized materials were determined in the context of light emission and the lasing potential of such fibers. Based on the obtained results, it can be concluded that the presented materials can be used to manufacture polymer optical fibers that can potentially offer attractive luminescent and laser features.

## 2. Materials and Methods

The complexes of europium(III) and terbium(III) with 2,6-dichlorobenzoic acid (ALDRICH) were obtained in the reaction of lanthanide(III) chloride (Alfa Aesar) with an organic acid. The aqueous solution of dissolved 2,6-dichlorobenzoic acid (50 mL, 3 mmol) was added dropwise to the aqueous solution of lanthanide(III) chloride (50 mL, 1 mmol) heated to 80 °C. Next, trimethylamine (TEA) was added dropwise to the solution of substrates. The addition of TEA allowed for better deprotonation of the acid and coordination of metal ions. As the pH rose to approximately 5.5, crystals of the complexes immediately formed; they were filtered off, washed with a small amount of water, and dried at room temperature.

The compositions consisting of the Eu:2,6-DClB or Tb:2,6-DClB complex and methyl methacrylate (MMA, ALDRICH, Warsaw, Poland), as the polymer matrix, as well as an initiator (dibenzoyl peroxide, BPO) (ALDRICH, Warsaw, Poland) and thioglycolic acid, as the chain transfer agent (ALDRICH, Warsaw, Poland), were finally obtained. In their preparation, a constant concentration of the initiator (0.4 wt.%), chain transfer agent (0.8 wt.%), and dopants (1 wt.%) were applied (Table 1).

After mixing the abovementioned ingredients, all of the compositions were prepolymerized and, subsequently, the appropriate curing process was applied. The thermal polymerization was carried out in the test tube with an inner diameter of 14 mm. The process was carried out in an oven under vacuum conditions. The temperature was increased gradually from 25 to 110 °C over 2 days until solidification was fulfilled.

The attenuated total reflectance-Fourier transform infrared spectra (ATR/FT-IR) spectra were recorded using a Nicolet 6700 spectrophotometer equipped with the diamond Smart iTR accessory in the range of 4000–600 cm^−1^.

Single-crystal diffraction data for Eu(III) and Tb(III) 2,6-dichlorobenzoates were collected at 293 K on an Xcalibur CCD diffractometer (Oxford Diffraction, Oxford, UK) with the graphite-monochromated Mo Kα radiation (α = 0.71073 Å). The programs CrysAlis CCD and CrysAlis Red (CrysAlis PRO) [28] were used for data collection, cell refinement, and data reduction. A multi-scan absorption correction was applied. The structures were solved by direct methods using SHELXS-97 and refined by full-matrix least squares on F2 using SHELXL-97 [29].

Thermal analysis was carried out on a STA 449 Jupiter F1, (Netzsch, Selb, Germany) under the following operational conditions: heating rate 10 °C min^−1^, a dynamic atmosphere of helium (50 mL min^−1^) in the temperature range of 25–600 °C, sample mass of approximately 5 mg, and sensor thermocouple type S TG-DSC. As a reference, an empty Al crucible was used. The identification of the gas composition emitted during the heat increase was performed using the quadrupole mass spectrometer QMS 403 C Aëolos (Netzsch, Selb, Germany) coupled online to the STA instrument. The mass spectrometer was connected online to the STA instrument by a quartz capillary heated to 300 °C. The QMS was operated using an electron impact ionizer with the energy 70 eV. The measurements were performed in the scan mode for *m/z*, where m is the mass of molecule and z is a charge of the molecule in the electron charge units in a range from 10 to 200 amu, allowing for the identification of all possible volatile particles produced during the decomposition.

For the characterization of luminescent properties of developed materials, the modular PTI QuantaMaster-based spectrofluorometer was used (HORIBA Scientific, Ontario, Canada), equipped in double monochromators in excitation and emission paths, CW, and pulse broadband lamps (200–2000 nm) as the excitation light sources. An ultra-sensitive PMT detector was used for recording the excitation and emission spectra as well as luminescence kinetics profiles. The optical measurements were taken at room temperature, and all spectra were corrected for spectral characteristics of the detector’s response.

## 3. Results and Discussion

### 3.1. Structural Characterization of Dopants

**Figure 1:**
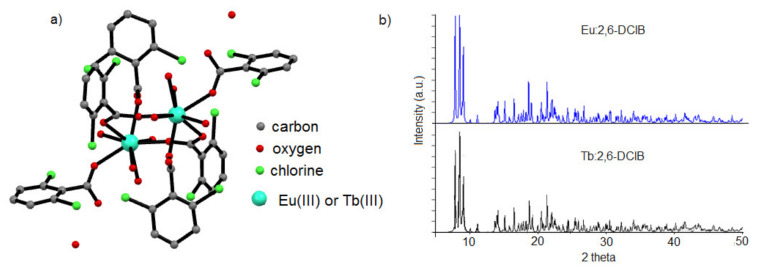
(**a**) Structural characterization of the dopants: europium(III) and terbium(III) complexes with 2,6-dichlorobenzoic acid (hydrogen atoms were omitted for figure clarity); (**b**) XRD patterns of lanthanide complexes.

The europium(III) and terbium(III) ions form with 2,6-dichlorobenzoic acid (H2,6DClB) complexes of the general formula [Ln_2_(C_7_H_3_O_2_Cl_2_)(H_2_O)_8_]·2H_2_O, where Ln = Eu(III) and Tb(III); C_7_H_3_O_2_Cl_2−_ = 2,6DClB. The abbreviations used henceforth for the complexes in the manuscript are Eu:2,6-DClB and Tb:2,6-DClB. Single-crystal X-ray analysis reveals that both complexes crystallized in the triclinic system and space group P$\bar{1}$. Recorded unit cell parameters for the investigated complexes (for Eu2,6DClB: a = 11.2184(5) Å; b = 12.5908(5) Å; c = 12.9021(5) Å; α = 116.610(4)°; β = 114.438(4)°; γ = 95.928(3)° and V = 1471 Å^3^; Tb2,6DClB: a = 11.1572(6) Å; b = 12.6195(7) Å; c = 12.8379(7) Å; α = 111.679(5)°; β = 12.6195(7)°; γ = 95.810(4)° and V = 1460.24 Å^3^) perfectly fit with the data published previously for Ce(III), Pr(III), Nd(III), Tb(III), and Dy(III) 2,6-dichlorobenzoates [30]. Metal centers in the dimeric complexes were bonded by two bridging carboxylate groups from 2,6-dichlorobenzoate ligands (Figure 1).

The octacoordinated Ln(III) ions were bonded by four carboxylate oxygen atoms from four organic ligands and four aqua ligands. Additionally, two water molecules were in the outer coordination sphere of lanthanide (ions).

**Figure 2:**
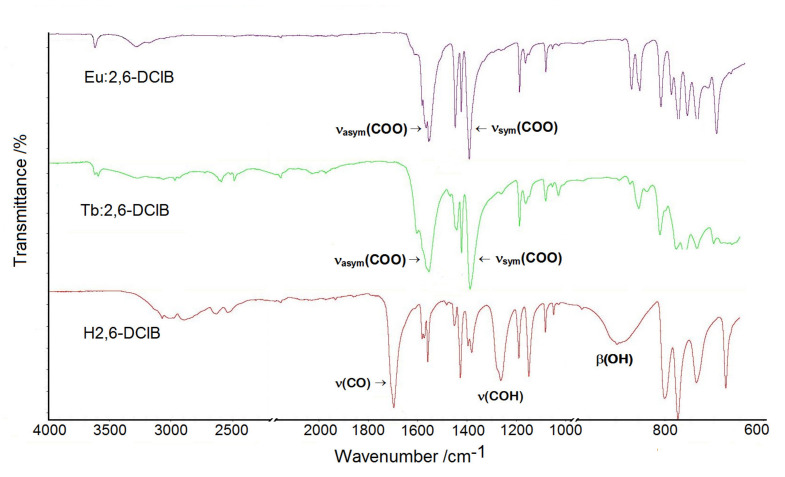
The ATR/FT-IR spectra of free 2,6-dichlorobenzoic acid and its complexes with Eu(III) and Tb(III).

The ATR/FT-IR spectra of lanthanide 2,6-dichlorobenzoates showed sharp bands at approximately 3620 cm^−1^ assigned to the υ(OH) from water molecules that were not engaged in hydrogen bonds. At a lower wavenumber range of 3600–2600 cm^−1^, broad bands appeared with several submaxima associated with stretching vibrations of C–H groups as well as O–H groups of water molecules arranged in hydrogen bonds. The infrared spectrum of free 2,6-dichlorobenzoic acid was dominated by the characteristic vibrations of carboxylic group which gave bands at 1704, 1269, and 900 cm^−1^ due to the υ(CO), υ(COH), and β(OH) vibrations. These bands disappeared in the infrared spectra of metal complexes due to the coordination process. The most relevant bands of lanthanide complexes appeared at 1572/1560 and 1397/1385 cm^−1^ as a result of stretching asymmetric (υ_asym_) and symmetric (υ_sym_) vibrations of carboxylate groups (Figure 2).

Splitting of the υ_asym_(COO) bands is a diagnostic for different coordination modes (monodentate and bidentate bridging) of COO groups in the structures of complexes. The remaining bands observed on the infrared spectra were assigned to the stretching and deformations vibrations of C_Ar_C_Ar_, C_Ar_-H, C_Ar_-Cl groups as well as in-plane and out-of-plane vibrations of the benzene ring [31,32].

### 3.2. Thermal Analysis of Lanthanide Complexes

**Figure 3:**
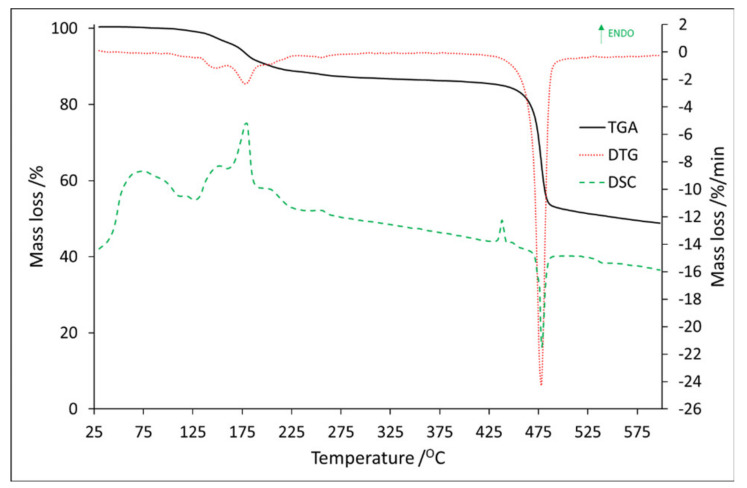
TG/DTG–DSC curves for Tb:2,6-DClB.

Full thermal analysis of Eu:2,6-DClB, sample No. 1, was reported in our previous article [33]. The TG/DTG–DSC curves of Tb:2,6-DClB, sample No. 2, are shown in Figure 3, while all thermal data collected for sample No. 1 and No. 2 are listed in Table 2.

Based on the DTG curves it can be seen that the decomposition process for sample No. 2 can be divided into seven steps. The first five steps occurred from 100.0 °C to 275.0 °C with a mass loss of 1.0 wt.%, 2.54 wt.%, 6.0 wt.%, 1.7 wt.%, and 1.3 wt.%, respectively.

**Figure 4:**
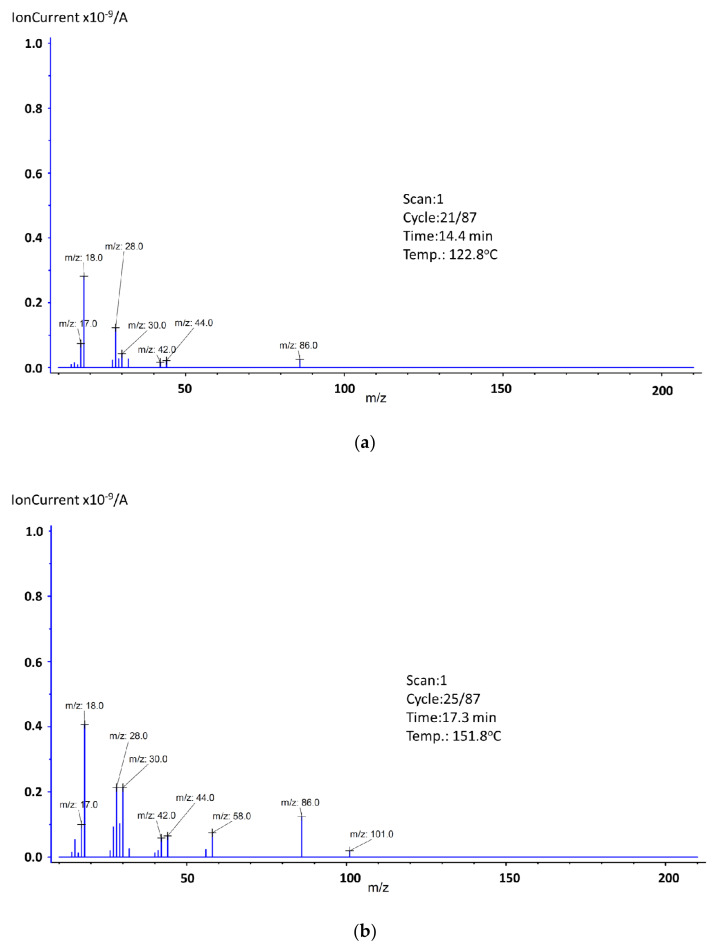
Mass spectra of Tb2,6DClB at endothermic maxima: (**a**) 122.8 °C, (**b**) 151.8 °C. Some shifts compared to the DSC temperature value placed in Table 2 are observed because of the apparatus response time.

In addition, at this range (with the little shift to the higher temperatures), five endothermic peaks were observed with the maximal values at 118.6 °C, 151.7 °C, 179.4 °C, 205.0 °C, and 254.4 °C (The graphs showing the ion distribution for the last 3 temperatures can be found in the additional materials (Figure S1)). The mass spectra recorded at these maxima are shown in Figure 4.

In the first step that occurred between 100.2 and 132.6 °C, mass spectra confirmed the release of water molecules due to the presence of ions *m/z*: 16, 17, and 18 (Figure 4a). In addition to the water, the emission of organic volatile decomposition products was also detected. Based on the ion fragment values and the synthesis process, we suspect the presence of triethylamine. Ion CH_2_=N^+^H_2_ was visible as the value *m*/*z*: 30, 29, and 28. Ion 86 (M^+^ − 15), which is related to the presence of CH_3_CH_2_N^+^(=CH_2_)CH_2_CH_3_, arising as a result of an α-cleavage. If it comes to ion *m*/*z*: 44, it can be associated with the presence of a carboxylic group, but since we observed ion *m*/*z*: 42, and there were no others that could confirm the degradation of benzoic acid, we assumed that those were also the confirmation of the triethylamine presence [34]. The second step occurred in the temperature range of 132.6–159.2 °C, where 2.5 wt.% of mass loss was detected. Based on the MS spectra, the presence of water and triethylamine was observed (Figure 4b). Compared to Figure 4a, on this spectrum two new ions were detected, *m*/*z*: 58 and 101, both confirming the triethylamine presence. The *m/z*: 101 is a molecular peak (M^+^), and *m/z*: 58 is a fragment CH_3_N^+^(=CH_2_)CH_3_. The third step fell in the temperature ranging from 159.2 to 201.0 °C, with the 6.0 wt.% of mass loss. Based on the MS spectra, it can be concluded that all previously occurring ions were still present, but their signals’ intensity had increased. The same situation, but with decreased signal intensity, was for the fourth (range: 201.0–231.3 °C) and the fifth (range: 231.3–275.0 °C) step. The sixth stage was also related to the endothermic process. It fell in the temperature range 423.7–446.5 °C, with a maximum at 437.7 °C (The graph can be found in the Supplementary Materials (Figure S2)). The recorded mass loss at this range was 1.1 wt.%.

**Figure 5:**
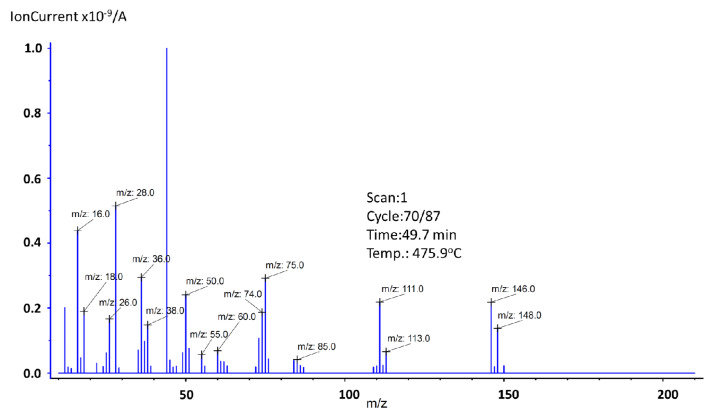
Mass spectra of Tb2,6DClB at the exothermic maximum 478.5 °C.

Besides water fragments (*m*/*z*: 16, 17, 18, and 36) and, possibly, triethylamine residues (*m/z*: 26, 28, and 44), no new ions were observed. It can be concluded that the dehydrated form of compound Tb(dclb)_3_ (after removing triethylamine) is thermally stable up to approximately 446.5 °C. Further heating results in the decomposition of the anhydrous form of the complex (the seventh step—exothermic effect) and thermal decomposition of the organic ligand. In the final solid of the complex, heated in an inert atmosphere, most probably a mixture of carbon, TbOCl, and Tb_4_O_7_ is formed. The residual mass was recorded as 48.8 wt.%. The maximum exothermic effect was at 478.5 °C, and the mass spectrum recorded at this temperature is shown in Figure 5.

In addition to the abovementioned, there were also traces of other ions recorded. In the spectrum of a compound containing a single chlorine atom, the isotope peak of the molecular ion (M^+^ + 2) showed an intensity of 32% compared to the M^+^ ion peak, which results from the typical presence of the chlorine isotopes ^35^Cl and ^37^Cl in nature in such a ratio [35]. The same relationship also applies to the fragmentation ions containing a chlorine atom. Ion *m*/*z*: 111, which is a fragmentation peak, is related to the presence of [C_6_H_4_^35^Cl]^+^ and that means that ion *m*/*z*: 113 will also be observed as a [C_6_H_4_^37^Cl]^+^. Additionally, its intensity is about one-third of the peak height at *m/z*: 111. The same situation can be observed for fragmentation peak *m/z*: 85 [C_4_H_2_^35^Cl]^+^ and *m*/*z*: 87 [C_4_H_2_^37^Cl]^+^. The greater number of chlorine atoms in the ion causes additional isotope peaks (M^+^ + 4), (M^+^ + 6), etc. For our substance, which has two chlorine atoms in its structure, one fragmentation peak (*m/z*: 146, [C_6_H_4_^35^Cl_2_]^+^) and two isotope peaks (*m*/*z*: 148, [C_6_H_4_^35^Cl^37^Cl]^+^) (*m*/*z*: 150, [C_6_H_4_^37^Cl_2_]^+^) were observed. In this case, the intensity of the first isotope peak should be approximately 65% and the second approximately 10% of the peak height at *m*/*z*: 146, which was preserved in our analysis [35,36]. Additionally, the ions *m*/*z*: 38, 50, 55, 60, and 75 arose as a result of the fragmentation of the phenolic group.

### 3.3. Thermal Analysis of Hybrid Composites

**Figure 6:**
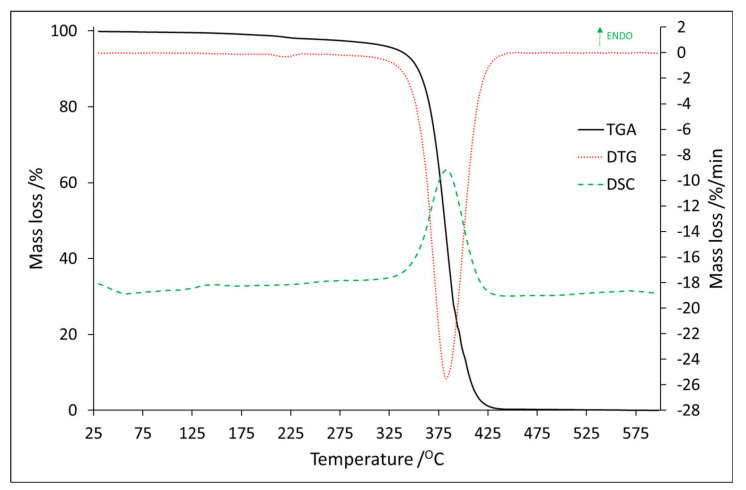
The TG/DTG–DSC curves for Eu:2,6-DClB/PMMA.

Full thermal and spectroscopic analysis of the PMMA polymer matrix was presented in the previous work [27]. The TGA/DTG and DSC results for Eu2,6DClB/PMMA (sample No.3) are shown in Figure 6, and all thermal properties are summarized in Table 3.

It can be observed that, for sample No. 3, the mass loss can be divided into three steps, and all of them are endothermic, even the last one, which is certainly related to thermal decomposition. In comparison to thermal properties of Eu:2,6-DClB and Tb:2,6-DClB, where the thermal decomposition was sufficiently exothermic, after combining dopants with the polymer matrix, thermolysis required heat to break the bonds. The value of mass loss for these steps was 0.6 wt.%, 0.8 wt.%, and 96.7 wt.%, respectively.

**Figure 7:**
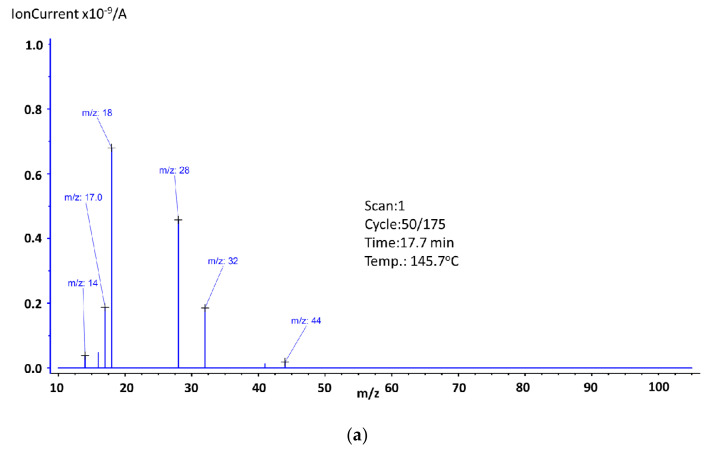

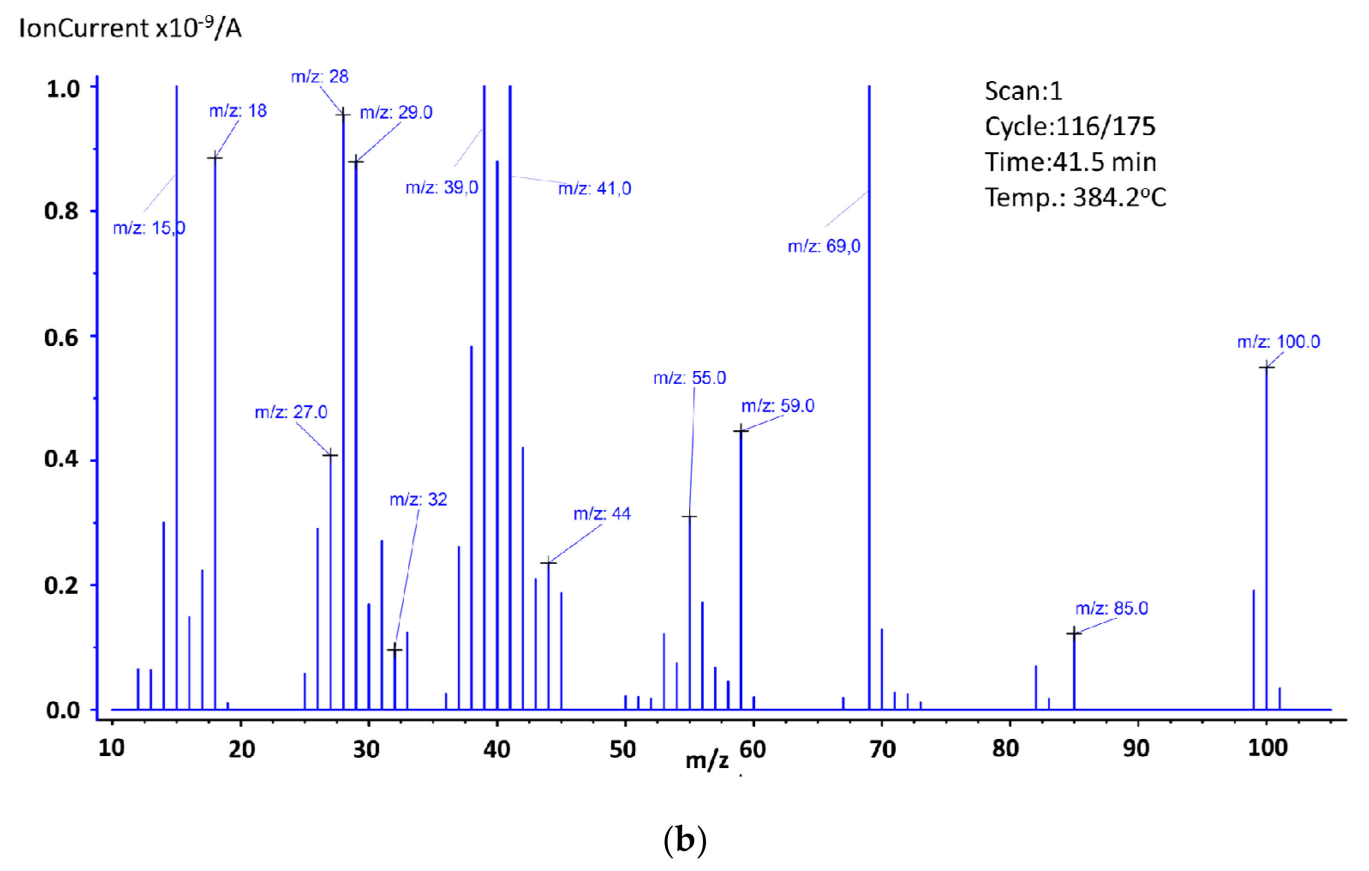
Mass spectra of Eu2,6DClB/PMMA at endothermic maxim: (**a**) 145.7 °C, (**b**) 384.2 °C. Some shifts compared to the DSC temperature value shown in Table 3 are observed because of the apparatus response time.

For better understanding of the reason for the mass loss and the endothermic effects, mass spectroscopy spectra were recorded at the maxima of the endothermic effects and are shown in Figure 7.

As it was already mentioned, three steps of mass loss can be observed. The first one, which occurred between 123.3 °C and 190.0 °C, was related to the presence of water, which was confirmed by mass spectroscopy, where ions *m*/*z*: 16, 17, and 18 were recorded (Figure 7a). In this range, ions *m*/*z*: 14 ([CH_2_]^+^), *m*/*z*: 28 (since there were no other ions that could confirm the presence of trimethylamine or vinyl group, we assumed this ion was related to the presence of [CO]^+^), *m*/*z*: 32 (this ion could be responsible for the presence of the methanol molecules that formed during the decomposition of PMMA, but since there were no other ions to support this theory, we assumed it was O_2_), and *m*/*z*: 44 ([COO]^+^) were also recorded. It should be noted that the mass loss registered for this temperature region was small (0.6 wt.%), which means that since an endothermic effect was observed, we can assume water evaporation and other ions came from surface-adsorbed oxygen or carbon dioxide molecules. The second step fell in the region 201.3–240.1 °C. The endothermic effect for this mass loss shifted towards higher temperatures, and its maximum was noted at 261.3 °C (The graph can be found in the Supplementary Materials (Figure S3)). Based on obtained results, we can confirm the presence of the very same ions as in the first mass loss step. New ion *m*/*z*: 15 was related to the presence of [CH_3_]^+^ and ion *m*/*z*: 41 to the presence of [C_3_H_5_]^+^. The last mass loss step was observed in the temperature range of 298.0–454.2 °C. As it was mentioned, in this range a proper decomposition of the studied substance takes place (96.7 wt.% of mass loss), which is accompanied by an endothermic energy effect. These results indicate that sample No. 3 was characterized by very good thermal stability up to a temperature of almost 300.0 °C. To determine the possible molecules’ fragments formed during the decomposition of the studied substance, at the maximum of the endothermic effect, the mass spectrum was recorded (Figure 7b). Based on obtained results, the presence of the following ions was identified: *m*/*z*: 14,15 (methylene and methyl group), *m*/*z*: 16,17,18 (water, hydroxyl group), *m*/*z*: 26, 27, and 28 (vinyl group), *m*/*z*: 29 (ethylene group), *m*/*z*: 30, 31, 32, and 33 (methanol). The ion *m*/*z*: 57 ([C_4_H_9_]^+^] very easily splits off methane molecule, thus forming the propene ion (*m*/*z*: 41 or 42) which then transforms into the more stable cyclopropene ion (*m*/*z*: 39). Other identified ions were *m*/*z*: 40 ([C_3_H_4_]^+^) and *m*/*z*: 43, 44, and 45, which are associated with carboxyl and acetyl groups. The ion *m*/*z*: 100 is a molecular peak (M^+^), which means that *m*/*z*: 101 was associated with the presence of the ^13^C isotope carbon, and *m*/*z*: 99 was formed by the detachment of a hydrogen atom. The ion *m*/*z*: 85 (M^+^-15), which is related to the presence of CH_2_=C(CH_3_)C(O)=O, arose as a result of an α-cleavage. The ion *m*/*z*: 69 is related to the presence of CH_2_C(CH_3_)CO, which results from the loss of the methoxy group from the methyl methacrylate molecule. During the PMMA decomposition, substances, such as 2-methyl-2-butene, 2-methyl-1-butene, 2-methylpropanal, methyl acrylate, methyl propionate, methyl isobutyrate, were formed and ions *m*/*z*: 50, 51, 52, 53, 54, 55, 56, 58, 59, 60, 70, 71, 72, 73, 82, and 83 confirm this statement [37].

**Figure 8:**
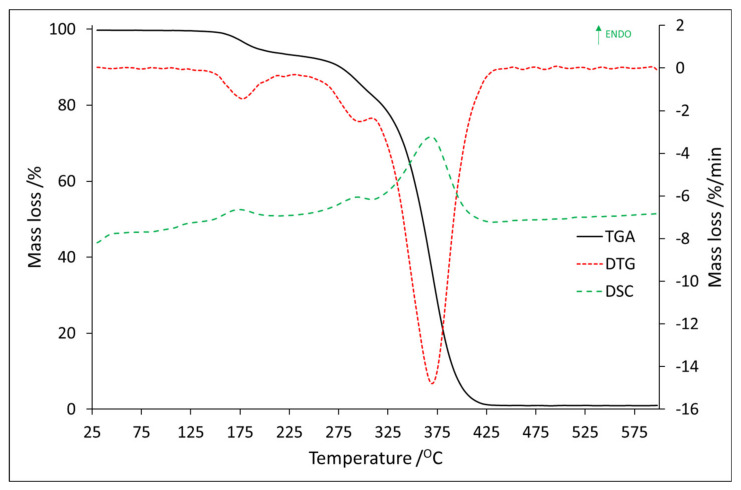
The TG/DTG–DSC curves for Tb:2,6-DClB/PMMA.

The TGA/DTG and DSC results for Tb:2,6-DClB/PMMA (sample No. 4) are shown in Figure 8, and all thermal properties are summarized in Table 3.

**Figure 9:**
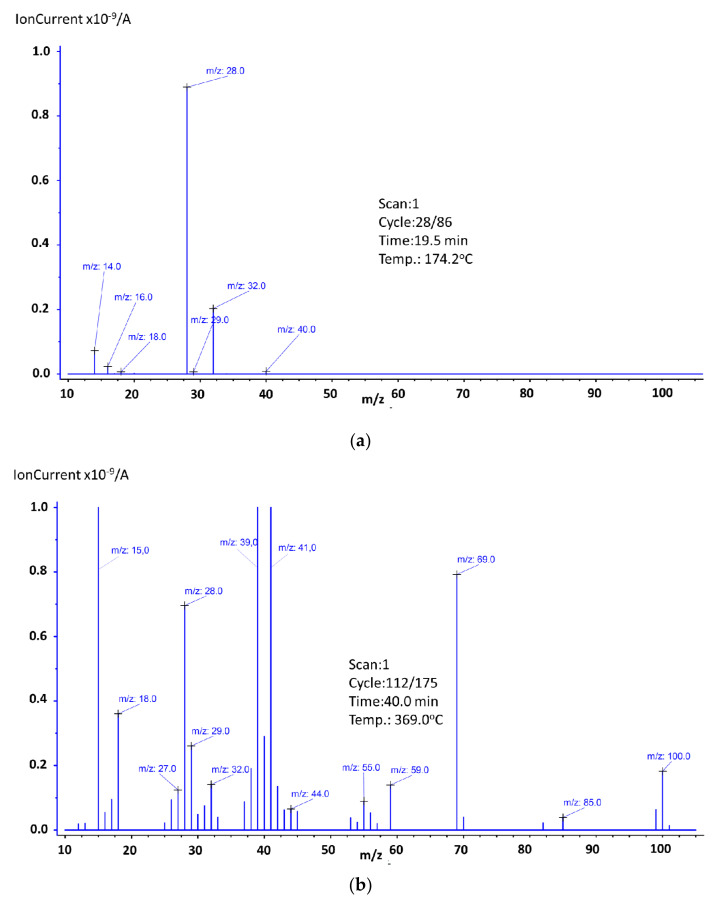
Mass spectra of Tb:2,6-DClB/PMMA at endothermic maxima: (**a**) 174.2 °C; (**b**) 368.8 °C. Some shifts compared to the DSC temperature value shown in Table 3 were observed because of the apparatus response time.

Similar to sample No. 3, sample No. 4 was characterized by three mass loss steps. They were recorded in the following temperature ranges: 136.0–230.0 °C, 240.8–310.5 °C, and 310.5–442.2 °C, with a mass loss of 6.3 wt.%, 10.5 wt.%, and 81.3 wt.%, respectively. Three endothermic effects were noted in these ranges, and Figure 9 shows the mass spectra corresponding to their maxima.

For losing mass in the first stage ions *m*/*z*: 16 and 17 (water evaporation) were responsible (Figure 9a). Since ions *m*/*z*: 14, 28, and 29 were recorded, we need to take into account that some triethylamine residues could be present in the tested material. This has to be considered, in addition, because of the value of the mass loss of 6.3 wt.%. It should therefore not be assumed that it results only from the evaporation of surface-bound substances. The presence of ion *m*/*z*: 39 is rather surprising. Due to the absence of other ions indicating the onset of thermal decomposition, it must be assumed to be just contamination, most likely a residue in the quartz tube from a previous analysis where the sample was thermally decomposed. The presence of ion *m*/*z*: 32 needs to be considered as oxygen. The mass spectra recorded for the second mass loss stage was the same as it was for the first stage (The graph can be found in the Supplementary Materials (Figure S4)). Based on the obtained results, it might be stated that within the discussed temperature range, there was no thermal decomposition observed. As it was mentioned, the last mass loss step fell in the range 310.5–442.2 °C. Based on the mass loss value (81.3 wt.%) and the mass spectra (Figure 9b), it can be concluded that in this range a proper decomposition took place. It should then be stated that sample No. 4 was thermally stable up to approximately 310 °C. The maximum endothermic effect was observed at 369.0 °C, and the recorded mass spectrum in this temperature is shown in Figure 9b. The presence of the following ions was confirmed: *m*/*z*: 15 (methylene group), *m*/*z*: 16, 17, and 18 (water, hydroxyl group), *m*/*z*: 26, 27, 28, and 29 (vinyl and ethylene group), *m*/*z*: 30, 31, 32, and33 (methanol), *m*/*z*: 38, (fragment of phenolic group), *m*/*z*: 40 ([C_3_H_4_]^+^), *m*/*z*: 43, 44, and 45 (carboxyl and the acetyl groups), and *m*/*z*: 59 (COOCH_3_). The ion *m*/*z*: 57 ([C_4_H_9_]^+^) by splitting off methane molecule forms the propene ion (*m*/*z*: 41 or 42) which then transforms into cyclopropene ion (*m*/*z*: 39). Ion 85 (M^+^-15) arose as a result of α-cleavage, is related to the presence of CH_2_=C(CH_3_)C(O)=O. The loss of the methoxy group from the molecular peak—methyl methacrylate (ion *m*/*z*: 99, 100, and 101)—contributed to the formation of the ion 69 (CH_2_C(CH_3_)CO). Ions *m*/*z*: 53, 54, 55, and 56 were responsible for the presence of [C_4_H_5_]^+^, [C_4_H_6_]^+^, [C_4_H_7_]^+^, and [C_4_H_8_]^+^ and formed as a result of the thermal decomposition of PMMA particles [37].

### 3.4. Luminescent Properties of Developed Complexes and Hybrid Materials

Apart from the thermal properties of the developed materials, critical from the point of view of transforming them into polymer optical fibers (POFs), the luminescent properties were also analyzed and discussed in the context of the development of optically active POFs, enabling not only the transmission of optical signals but potentially obtaining light emission, amplification, and lasing. To provide all these functionalities an active medium is a must, and one promising approach is to use the carboxylate complexes of lanthanide ions introduced into polymeric matrices. The most important challenge of this approach is to develop a lanthanide complex with sufficiently good luminescent properties and preserve these while introduced into the polymer host. Below, the results of our research are presented for two complexes, one with europium and the second with terbium ions, known for excellent luminescence in the red and green spectral range, respectively.

#### 3.4.1. Eu2,6DClB/PMMA

The first investigated complex, Eu:2,6-DClB, based on the europium(III) ions, exhibited luminescence in the long-wavelength (orange–red) part of the visible spectral range, typical for the majority of Eu^3+^ doped materials. For exciting this luminescence, the UV radiation has to be used at approximately 300 nm, which is, to some extent, inconvenient for excitation of the polymer fibers but enables in-depth optical characterization of emission properties of this material.

**Figure 10:**
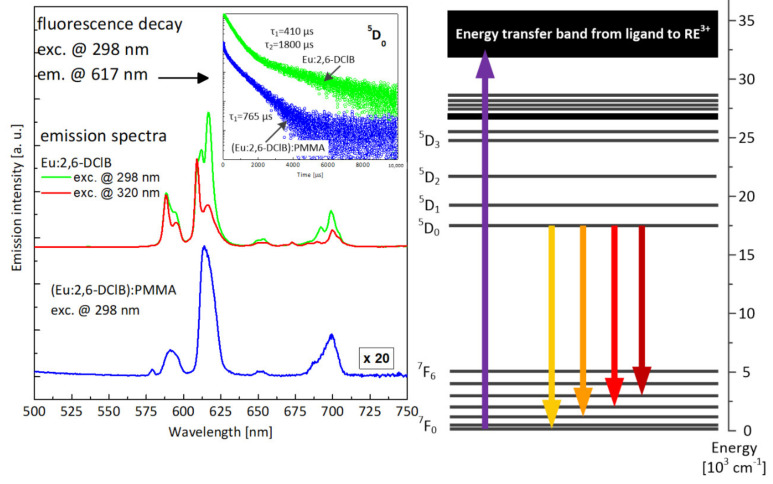
Comparison of luminescence spectra recorded for the Eu:2,6-DClB complex and the Eu2,6DClB/PMMA polymer composite (fluorescence decays for both materials shown in inset) together with the attribution of luminescent transition within the energy-level structure of the europium ion (on the right).

On the emission spectrum of Eu:2,6-DClB (Figure 10), there are clearly visible emission lines related to optical transitions from metastable ^5^D_0_ singlet to ^7^F_J_ multiplets. The dominant character had the line centered at approximately 613 nm, related with transition ^5^D_0_–^7^F_2_, which again is typical for trivalent europium-doped materials. It should be noted that the luminescence characteristics changed slightly depending on the excitation wavelength (red and green curves in Figure 10). The difference between the characteristics measured under excitation at wavelengths of 298 nm and 320 nm can be explained by emissions from europium ions located at different sites within the crystalline matrix of the complex. This was further confirmed by the doubly exponential character of the luminescence decay profile (see insert), with time constants equal to 410 µs and 1800 µs, respectively. The luminescence spectrum recorded for the composite sample (Eu:2,6-DClB/PMMA) showed that the hybrid composite kept the majority of the spectroscopic features of the europium(III) complex unchanged, although some influence of the polymer matrix, which interacted with active ions and was not fully isolated by a ligand, is clearly visible. It is possible to assume that during formation of hybrid materials, some water molecules from the coordination environment of europium(III) ions in metal complex were replaced by oxygen atoms from carbonyl groups of the polymeric matrix. Emission lines in the spectrum of Eu:2,6-DClB/PMMA are located within the same spectral ranges as in the case of the original complex, however, are considerably broadened. The luminescence decay profile is singly exponential (apart from the rapid decay seen in the first part of the curve and obviously related with the parasitic luminescence of PMMA host) with a time constant of 765 µs. This further confirms the influence of the polymer surroundings on europium ions.

**Figure 11:**
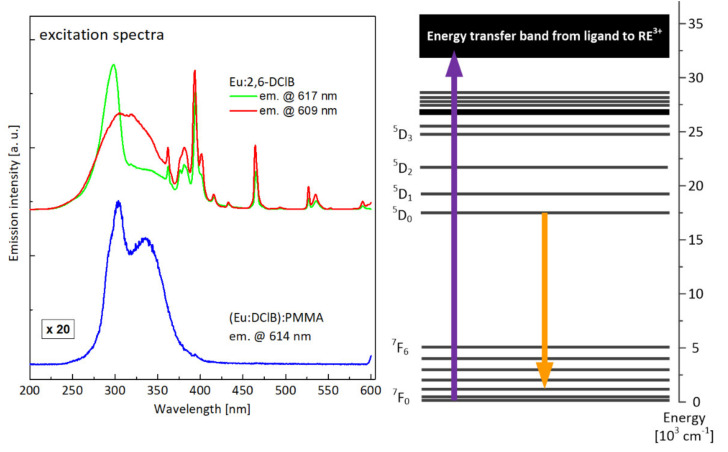
Comparison of the excitation characteristics recorded for the Eu:2,6-DClB complex and the Eu:2,6-DClB/PMMA polymer composite, together with the attribution of the monitored luminescent transition against the energy-level scheme of europium.

**Figure 12:**
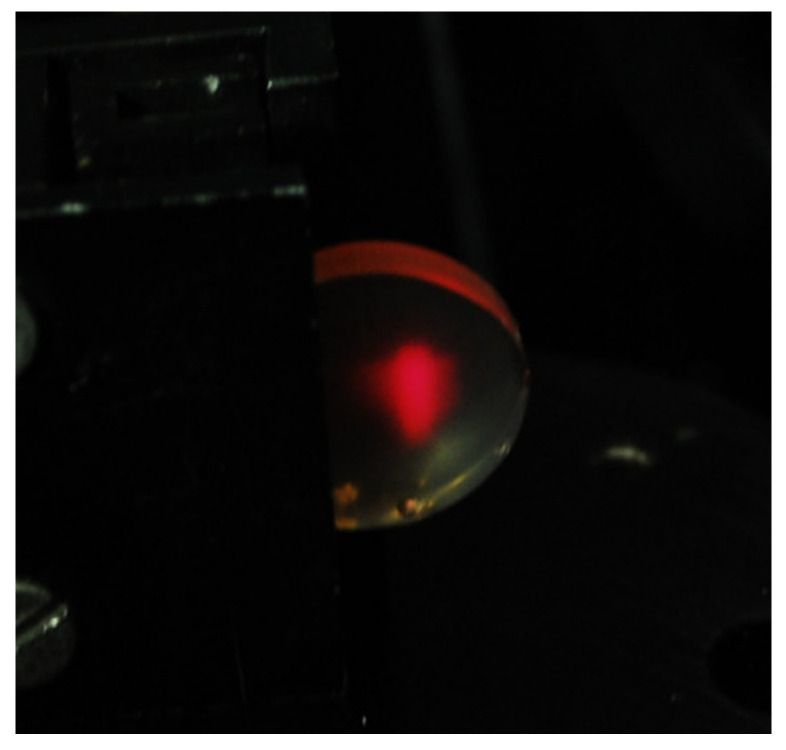
Photo of the Eu:2,6-DClB/PMMA in the measuring setup.

In Figure 11, excitation characteristics are presented, recorded for the most intense optical transitions. Broadband absorption bands visible on the M–O complexes spectra corresponded to the most efficient excitation of active ions via ligand (energy transfer from triplet state of the ligand, preceded by the absorption to the singlet state and intersystem crossing ISC to triplet state). Clearly visible, also, were narrow lines, related to the direct excitation of high lying energy levels of europium. The different character of excitation spectra, recorded for Eu:2,6-DClB while monitoring two different wavelengths of luminescence (i.e., 609 nm and 617 nm), confirms the notion that europium ions occupy different site positions in the M–O complex structure. In the case of a composite sample, the character of the excitation characteristics is different—the spectrum consists of a single broadband line with two maxima. The transitions corresponding to direct excitation of europium ions have vanished completely—the only way to provide the energy to active ions is to use UV excitation via coordinating ligands. In Figure 12, the photo of the hybrid polymer sample in the measuring setup is presented—the strong visible emission in the red part of the spectrum is visible.

#### 3.4.2. Tb:2,6-DClB/PMMA

The Tb:2,6-DClB complex with terbium(III) ions offers luminescence in the green part of the spectrum, observable, as in the case of the europium complex, under UV excitation at approximately 300 nm.

**Figure 13:**
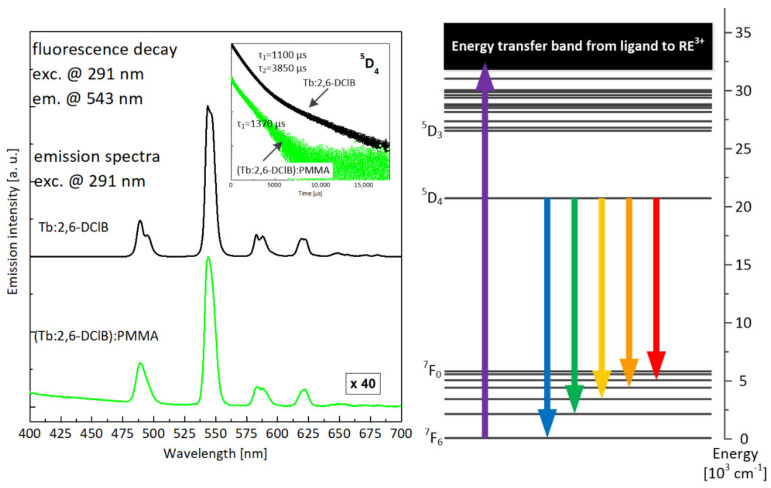
Comparison of luminescence spectra recorded for the Tb:2,6-DClB complex and the Tb:2,6-DClB/PMMA polymer composite (fluorescence decays for both materials shown in the inset) together with the attribution of the luminescent transition within the energy-level structure of terbium ion (on the right).

The luminescence spectrum of Tb:2,6-DClB, shown in Figure 13, exhibited the character typical of terbium-doped materials, with the dominant contribution of green emission. There were visible the four main emission lines, corresponding to optical transitions from metastable ^5^D_4_ level to ^7^F_J_ multiplets. The most intense was the line centered at approximately 543 nm, attributed to ^5^D_4_–^7^F_5_ transition, being typically dominant in Tb^3+^-doped materials. A doubly exponential luminescence decay profile (shown in the insert) suggests, similar to the case of the europium dopant, the presence of active ions in different site positions. The time constants were long (1100 µs and 3850 µs), favorable from the perspective of potential lasing properties of the investigated material. The luminescence spectrum recorded for composite hybrid material differed only slightly from this observed for terbium(III) complex. The spectral positions of all lines were the same, but the lines were slightly broadened. Single-exponential decay (green curve in the insert), with a time constant of 1370 µs, suggests stabilization of an active ion position, while a shortened lifetime was evidence of parasitic interactions with a polymer host, most probably via multi-phonon nonradiative depopulation of excited states.

**Figure 14:**
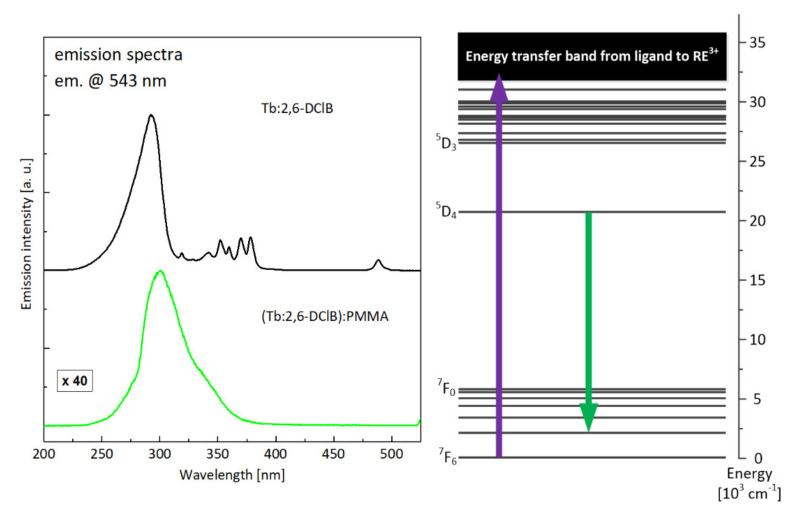
Comparison of excitation characteristics recorded for the Tb:2,6-DClB complex and the Tb:2,6-DClB/PMMA polymer composite, together with the attribution of the monitored luminescent transition against the energy-level scheme of terbium.

**Figure 15:**
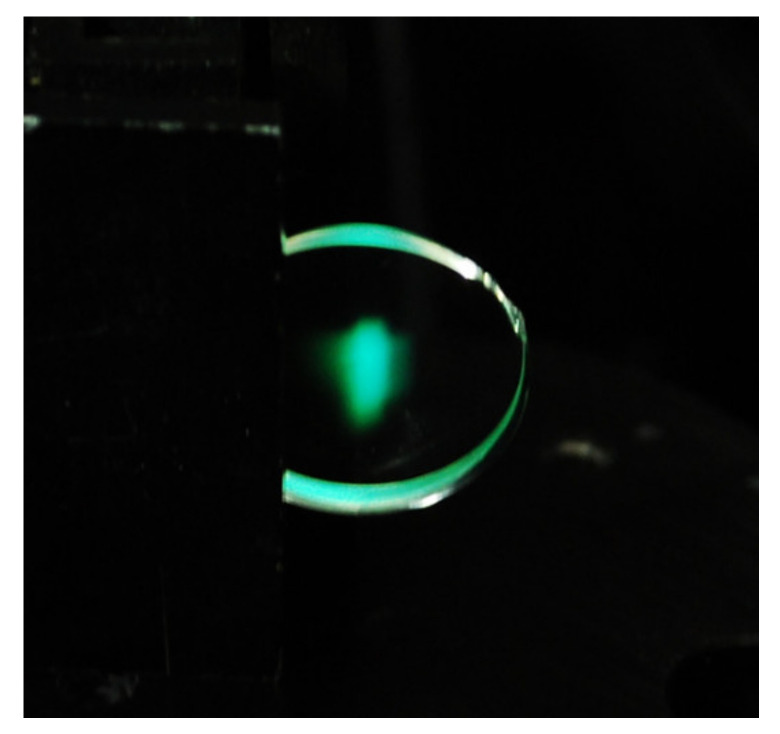
Photo of Tb2,6DClB/PMMA in the measuring setup.

Excitation spectra, compared in Figure 14 for the terbium(III) complex and polymer composite, differed mainly by the position of maximal intensity of absorption through ligand–ion energy transfer (which shifted towards longer wavelengths) and, similar to the europium ion case, by the lack of any optical transition to high energy levels of active ion. This means, that as in the case of europium, the only available mechanism of exciting terbium ions in investigated polymer composite is an energy transfer from ligand to active ion. In Figure 15, there is the photograph of the hybrid polymer sample activated with terbium ions taken during the measurements, showing the strong emission in the green part of the spectrum.

## 4. Conclusions

The europium(III) and terbium(III) 2,6-dichlorobenzoates were homogeneously dispersed in the PMMA matrix to form novel luminescent transparent hybrid materials, which were carefully characterized with respect to their thermal properties and luminescent features, both considered in the context of further processing into the form of polymer optical fibers.

Comparing the samples of Eu:2,6-DClB/PMMA and Tb:2,6-DClB/PMMA, it could be concluded that sample No. 4 showed a little higher thermal resistance than sample No.3. This statement is based on the information from the first recorded mass loss, which was found to be at 136.0 °C. However, it should always be taken into account the value of mass loss, and as we can see in Table 3 for sample No. 3, the value was 0.6 wt.% (as it was shown in Figure 7a, mass loss is related to the presence of water), and for sample No. 4, 6.3 wt.%. (as it was shown in Figure 9a, mass loss is related to the presence of water and trimethylamine). The second mass loss, in the case of both samples, was still not related to the decomposition of the sample, but only to the further release of the solvent molecules. It should be noted, however, that in the case of sample No. 4, it shifted considerably towards higher temperatures compared to sample No. 3. It is also worth noting that the addition of luminescent dopants did not deteriorate the thermal properties of the polymer matrix. As mentioned, the PMMA obtained in the Laboratory of Optical Fiber Technology was thermally stable up to approximately 270 °C. Based on the obtained results, the onset of decomposition for both hybrid materials was recorded around the temperature of 300 °C. This was confirmed by both thermal studies (Figure 6 and Figure 8) and mass spectra (Figure 7 and Figure 9). It can therefore be concluded that they are thermally stable at high temperatures, which is the most important conclusion of this part of the investigation.

The investigation of the luminescent properties of europium(III) and terbium(III) 2,6-dichlorobenzoates and resulting hybrid materials based on PMMA polymer showed, in turn, that both M–O complexes offer intense luminescence in the red and green spectral range depending on the lanthanide ion, the character of which doesn’t differ from the other organic and inorganic materials. The most important conclusion, however, is the fact that the luminescent properties of original complexes were very well preserved after being introduced into a polymer host. The resulting material had excellent transparency and had major emission properties that were practically unchanged, which, together with excellent thermal properties, might enable the development of an entirely new type of optically active polymer fibers, offering potentially light emission and, in a further perspective, amplification and lasing. This would, however, require solving many technological problems related to managing the luminescent features of the hybrid optical fibers, extending the choice of ions and range of emission wavelength as well as moving towards the more appropriate pumping schemes, while not utilizing ultraviolet radiation.

## Figures and Tables

**Table 1:** Experimental parameters of the syntheses—polymer matrix MMA.

Samples *	Dopant/wt.%		Initiator/wt.%	Chain Transfer Agent/wt.%	Methyl Methacrylate/wt.%
	Eu:2,6-DClB	Tb:2,6-DClB	-	-	-
Sample No. 1	100	-	0.0	0.0	0.0
Sample No. 2	-	100	0.0	0.0	0.0
Sample No. 3	1	-	0.4	0.8	97.8
Sample No. 4	-	1	0.4	0.8	97.8

* Sample mass is 25 g.

**Table 2:** TG/DTG–DSC data for sample No. 1 and 2.

Sample No.1	**Endothermic Peak**				
	DTG temperature range (°C)	DSC maximum value (°C)	TG mass loss (wt.%)		
	25–118	85	11.6		
	**Exothermic Peak**				
	374–500	460	40.5		
Sample No.2	**Endothermic Peak**				
	DTG temperature range (°C)	DSC maximum value (°C)		TG mass loss (wt.%)	
I	100.2–132.6	118.6		1.0	
II	132.6–159.2	151.7		2.5	
III	159.2–201.0	179.4		6.0	
IV	201.0–231.3	205.0		1.7	
V	231.3–275.0	254.4		1.3	
VI	423.7–446.5	437.7		1.1	
-	**Exothermic Peak**				
VII	446.5–509	478.5		32.3	

**Table 3:** TG/DTG–DSC data for samples No. 3 and 4.

Energy Effect		Thermal and Calorimetric Values	Sample No. 3	Sample No. 4
Endothermic peak	I	DTG Temperature range (°C)	123.3–190.0	136.0–230.0
		DSC maximum value (°C)	144.8	172.9
		TG mass loss (wt.%)	0.6	6.3
	II	DTG Temperature range (°C)	201.3–240.1	240.8–310.5
		DSC maximum value (°C)	261.3	293.3
		TG mass loss (wt.%)	0.8	10.5
	III	DTG Temperature range (°C)	298.0–454.2	310.5–442.2
		DSC maximum value (°C)	383.1	369.0
		TG mass loss (wt.%)	96.7	81.3

## Data Availability

The data underlying this article will be shared on reasonable request from the corresponding author.

## Supplementary Materials

The following are available online at https://www.mdpi.com/article/10.3390/ma14123156/s1, Figure S1: Mass spectra of Tb2,6DClB at endothermic maxima: (a) 181.0°C, (b) 202.7°C, (c) 253.0°C. Some shifts compared to the DSC temperature value placed in Table 2 are observed because of apparatus response time., Figure S2: Mass spectra of Tb2,6DClB, at endothermic maximum 437.7°C.; Figure S3. Mass spectra of Eu2,6DClB/PMMA at endothermic maximum 261.7°C. Some shifts compared to DSC temperature value placed in the Tab. 3 are observed because of apparatus response time. Figure S4. Mass spectra of Tb2,6DClB/PMMA at endothermic maximum 296.7°C. Some shifts compared to the DSC temperature value placed in Table 3 are observed because of apparatus response time.
