# Supplementary material for: Thermal, Spectroscopy and Luminescent Characterization of Hybrid PMMA/Lanthanide Complex Materials
Source: Materials (Basel). 2021 Jun 8;14(12):3156. doi: 10.3390/ma14123156 (PMC8228247; doi:10.3390/ma14123156)
Supplement: Supplementary file 1 [file materials-14-03156-s001.zip › materials-1219437-supplementary.pdf]

materials

Supplementary materials

Thermal, Spectroscopy and Luminescent Characterization of Hybrid PMMA/Lanthanide Complex Materials

Małgorzata Gil-Kowalczyk [1,*], Renata Łyszczek [2], Anna Jusza [3] and Ryszard Piramidowicz [3]

[1] Laboratory of Optical Fibers Technology, Faculty of Chemistry, Maria Curie-Sklodowska University, M. Curie-Sklodowska Sq. 5, 20-031 Lublin, Poland
[2] Department of General and Coordination Chemistry, Faculty of Chemistry, Maria Curie–Skłodowska University, M. Curie-Sklodowska Sq. 5, 20–031 Lublin, Poland; renata.lyszczek@poczta.umcs.lublin.pl
[3] Warsaw University of Technology, Institute of Microelectronics and Optoelectronics, Koszykowa 75, 00-662 Warsaw, Poland; anna.jusza@pw.edu.pl (A.J.); ryszard.piramidowicz@pw.edu.pl (R.P.)
* Correspondence: malgorzata.gil@poczta.umcs.lublin.pl

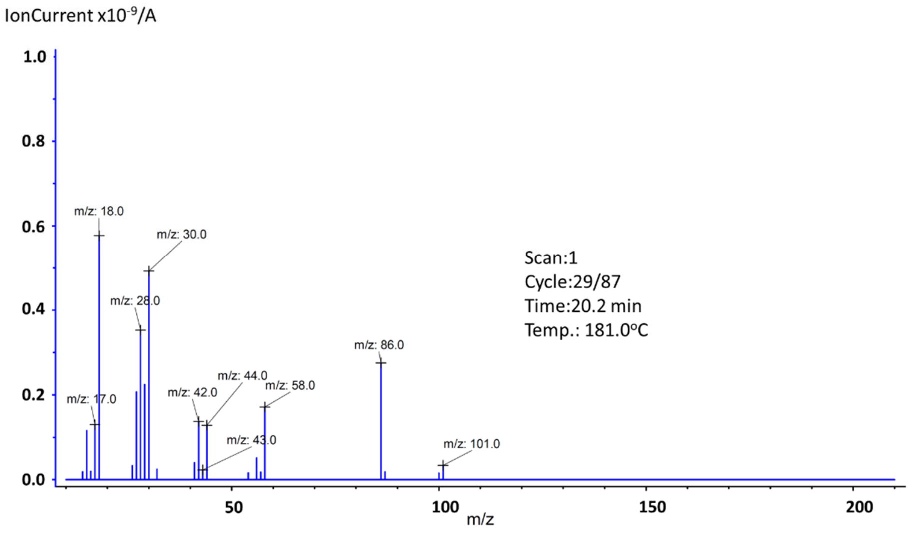

(**a**)

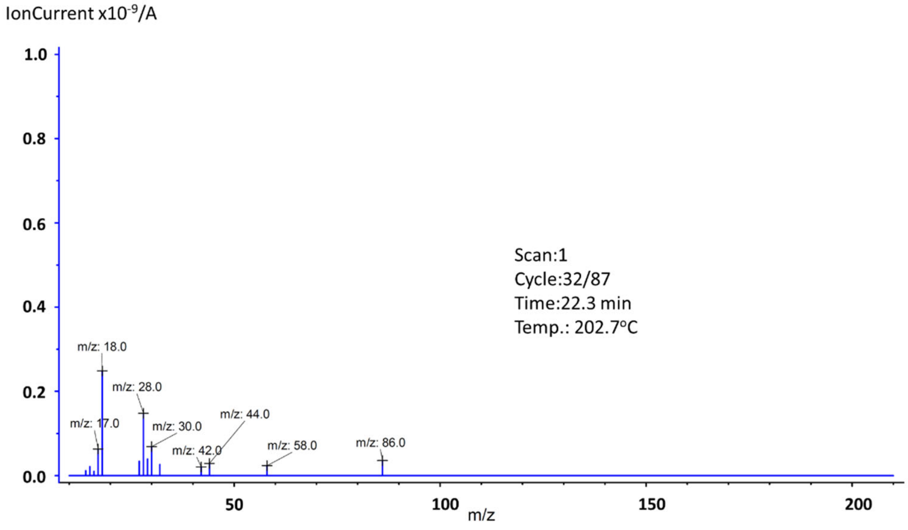

(**b**)

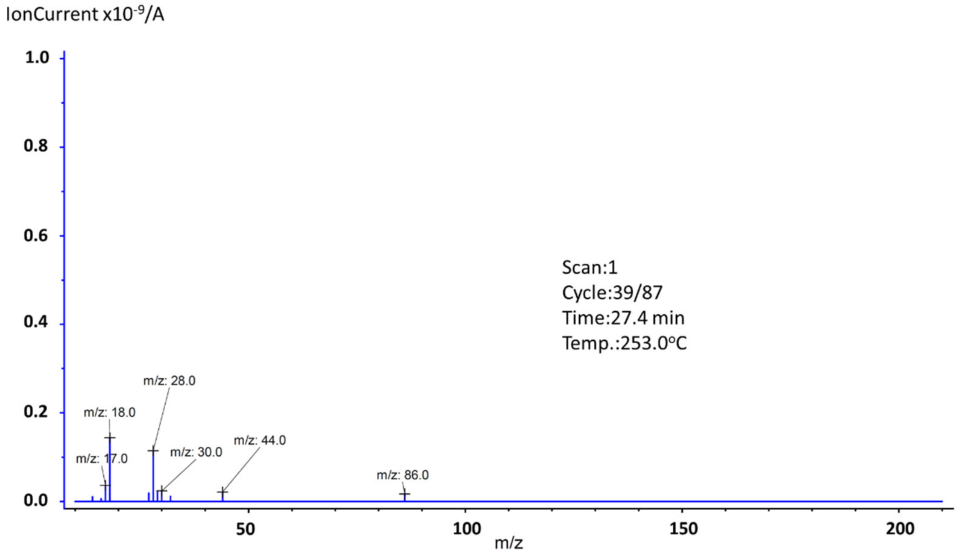

(**c**)

Figure S1. Mass spectra of Tb2,6DClB at endothermic maxima: (**a**) 181.0°C, (**b**) 202.7°C, (**c**) 253.0°C. Some shifts compared to the DSC temperature value placed in Table 2 are observed because of apparatus response time.

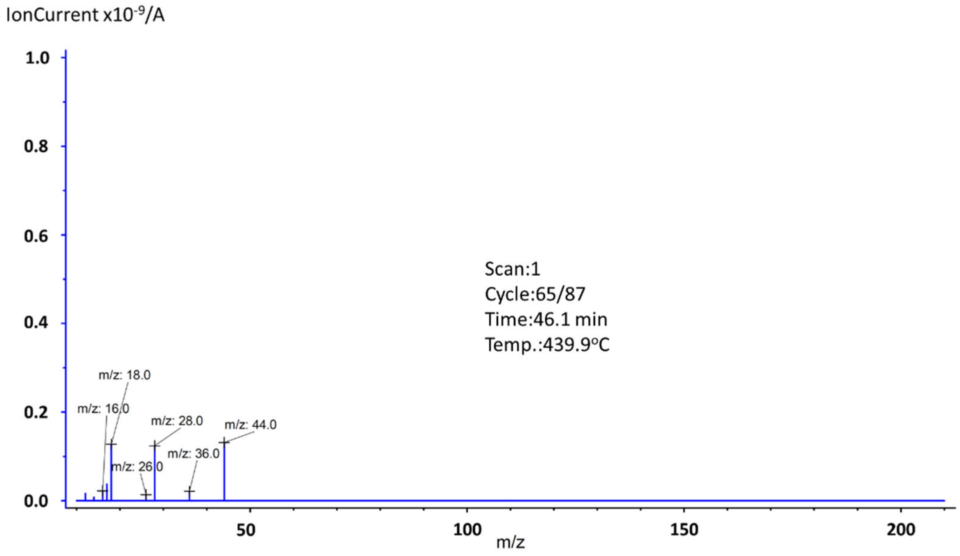

Figure S2. Mass spectra of Tb2,6DClB, at endothermic maximum 437.7°C.

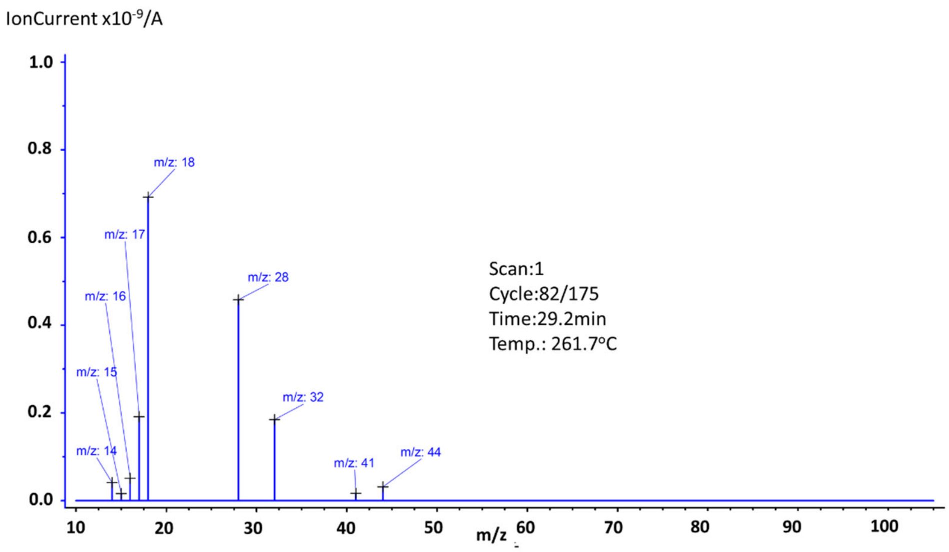

Figure S3. Mass spectra of Eu2,6DClB/PMMA at endothermic maximum 261.7°C. Some shifts compared to DSC temperature value placed in the Tab. 3 are observed because of apparatus response time

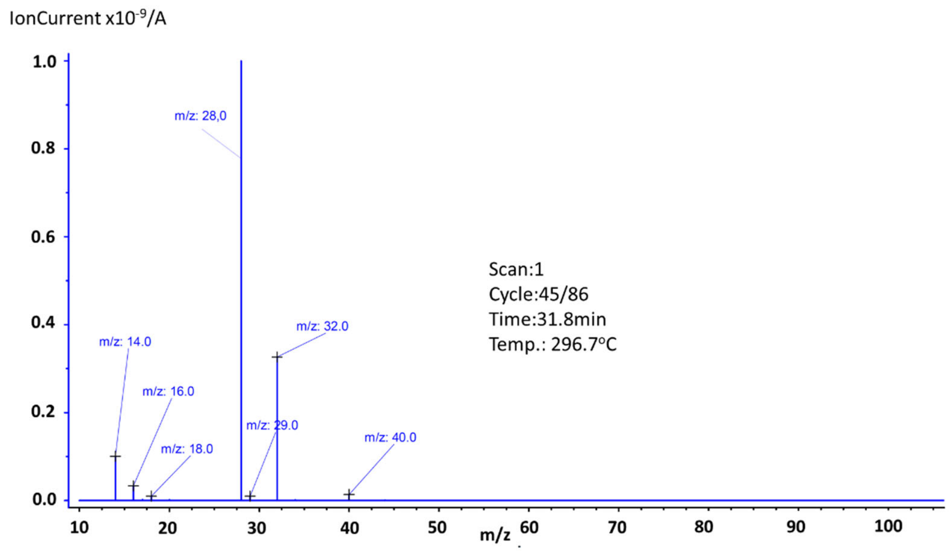

Figure S4. Mass spectra of Tb2,6DClB/PMMA at endothermic maximum 296.7°C. Some shifts compared to the DSC temperature value placed in Table 3 are observed because of apparatus response time.
